# Caffeic Acid Phenethyl Ester Protects against Experimental Autoimmune Encephalomyelitis by Regulating T Cell Activities

**DOI:** 10.1155/2020/7274342

**Published:** 2020-10-09

**Authors:** YiFan Zhou, Jingqi Wang, Yanyu Chang, Rui Li, Xiaobo Sun, Lisheng Peng, WenHua Zheng, Wei Qiu

**Affiliations:** ^1^Department of Neurology, The Third Affiliated Hospital of Sun Yat-sen University, Guangzhou, China; ^2^Institute of Translational Medicine, Faculty of Health Sciences, University of Macau, Taipa, Macau SAR, China

## Abstract

Multiple sclerosis (MS) is an autoimmune inflammatory disease of the central nervous system (CNS) characterized by progressive demyelination and disabling outcomes. CD4^+^ T cells are the most critical driving factor of relapsing MS, but little improvement has been noted upon deletion of the whole T cell population. Caffeic acid phenethyl ester (CAPE), one of the main active compounds of propolis, exhibits potent antitumour, anti-inflammatory, and antioxidant properties by suppressing nuclear factor-*κ*B (NF-*κ*B) transactivation. To investigate the therapeutic potential of CAPE in MS, we studied the effects of CAPE on cytokine levels, T cells, and NF-*κ*B activities and in an experimental MS animal model. The results showed that cerebrospinal fluid (CSF) from patients with relapsing MS is characterized by increased levels of proinflammatory cytokines/chemokines that preferentially skew towards T helper 1 (Th1) cytokines. *In vitro* studies demonstrated that CAPE not only inhibited T cell proliferation and activation but also effectively modulated T cell subsets. Under both Th0- and Th1-polarizing conditions, the proportion of CD4^+^IFN-*γ*^+^ cells was downregulated, while CD4^+^Foxp3^+^ cells were increased. Moreover, nuclear translocation of NF-*κ*B p65 was inhibited by CAPE. In a murine experimental autoimmune encephalomyelitis model, prophylactic treatment with CAPE significantly decreased the disease incidence and severity. Compared to the vehicle group, mice pretreated with CAPE showed diminished inflammatory cell infiltration, microglia/macrophage activation, and demyelination injury. Additionally, CAPE pretreatment reduced the level of Th1 cells in both spleen and the CNS and increased regulatory T cells (Tregs) in the CNS. In conclusion, our results highlight the potential merit of CAPE in suppressing T cell activity mainly through targeting the pathogenic Th1 lineage, which may be beneficial for MS treatment.

## 1. Introduction

Multiple sclerosis (MS) is an inflammatory demyelinating disease of the central nervous system (CNS) marked by repeated relapses and progressive disability. Although the exact mechanism of MS remains unclear, peripheral autoreactive T cells have been recognized to primarily drive relapsing MS, whereas B cells and CNS-resident cells contribute to progressive MS. [1] However, in a clinical trial, using monoclonal anti-CD4 antibody to selectively eliminate CD4^+^ T cells failed to reduce MS activity [2], which may be partially related to the fact that in addition to T helper 1 (Th1) and T helper 17 (Th17) cells, regulatory T cells (Tregs) also play a central role in MS pathogenesis by exerting potent anti-inflammatory activities. Therefore, regulating T cell activity rather than depleting the whole cell population would be more suitable. Recently, acute relapses in patients with MS were reported to be associated with aberrant nuclear factor-*κ*B (NF-*κ*B) gene expression in their T cells [1]. Since the NF-*κ*B signalling cascade is essential for cell proliferation, apoptosis, and immune responses, blocking this pathway may help to prevent MS exacerbation and progression.

Propolis has been widely used as a potential immunomodulatory agent. Increasing evidence has shown that many bioactive compounds of propolis can inhibit cytokine production and immune cell migration mainly by blocking the NF-*κ*B pathway [3, 4]. Caffeic acid phenethyl ester (CAPE) is one of the main active components of propolis, which has been reported to alleviate many inflammatory diseases such as allergic asthma, experimental autoimmune uveoretinitis, and sepsis as a specific NF-*κ*B inhibitor [5]. The chemical structure of CAPE is provided in the Supplementary Material (available here). In the present study, we aimed to investigate the impact of CAPE on T cells *in vitro* and to assess its therapeutic potential based on experimental autoimmune encephalomyelitis (EAE), which is a classic animal model for MS.

## 2. Materials and Methods

### 2.1. Materials

CAPE (purity ≥98%) was obtained from Nature Standard (Shanghai, China). Concanavalin A type IV (ConA), lipopolysaccharide (LPS), Percoll, Triton X-100, and dimethyl sulfoxide (DMSO) were purchased from Sigma-Aldrich. Recombinant mouse IL-12 (rIL-12; p70); purified Ultra-LEAF™ anti-mouse IL-4 antibody; purifiedLEAF™ anti-mouse CD3 antibody; purified LEAF™anti-mouse CD28 antibody; antibodies against mouse CD4, IL-17A, IFN-*γ*, Foxp3, CD25, and CD69; cell activation cocktail; brefeldin A solution; fixation/intracellular staining permeabilization wash buffer; and Foxp3 fixation/permeabilization buffer were obtained from BioLegend. RPMI 1640 medium, fetal bovine serum (FBS), and GlutaMAX were obtained from Gibco. Primary antibody against Iba-1 was purchased from Wako Pure Chemical Industries. Antibody against NF-*κ*B p65 was obtained from Santa Cruz, and myelin basic protein (MBP) was purchased from Proteintech. Alexa Fluor 546-conjugated goat anti-rabbit IgG (H + L) and Alexa Fluor 488-conjugated goat anti-mouse IgG (H + L) antibodies were purchased from Invitrogen. A haematoxylin-eosin (HE) staining kit and cell counting kit (CCK)-8 were purchased from Beyotime Biotechnology (Shanghai, China). Human XL Cytokine Array Kit was obtained from R&D.

### 2.2. Study Population and Cytokine/Chemokine Assays

Seven MS patients fulfilling the 2017 McDonald criteria were recruited between January 2018 and March 2018 [6]. Cerebrospinal fluid (CSF) was obtained within two weeks of acute attacks before acute treatment was started. Seven patients with noninflammatory diseases were included as controls (anxiety disorder, *n* = 2; tension headache, *n* = 2; and thrombosis of the intracranial venous sinus, *n* = 3). Patients with infections or other autoimmune diseases (e.g., asthma) were excluded from the study. CSF samples were centrifuged immediately and kept at -80°C until analysis. This study was approved by the Ethics Committee of the Third Affiliated Hospital of Sun Yat-sen University (Guangzhou, China) with written informed consent obtained from all participants. Cytokine/chemokine levels were analysed using a Proteome Profiler Human XL Cytokine Array Kit. Briefly, each membrane was placed in a 4-well multidish containing 2.0 ml of Array Buffer 6 in each well and blocked for 1 h. Then, Array Buffer 6 was aspirated from the wells, and prepared samples were added according to the protocol and incubated overnight at 4°C on a shaker. The membranes were washed three times with washing buffer, and a diluted detection antibody cocktail was added and incubated for 1 h at room temperature. After three washes, an HRP-conjugated secondary antibody was added to each well and incubated for 30 min at room temperature. Finally, Chemi Reagent Mix was added to the washed membranes, and then, the membranes were exposed to Tanon 5200.

### 2.3. Cell Preparation and Mitogen Stimulation

Female C57BL/6 mice (6-8 weeks old) were anaesthetized with pentobarbital and transcardially perfused with cold PBS. Spleens were removed and gently pressed through a 70-*μ*m nylon mesh. After centrifugation at 300 × g for 5 min, cells were treated with red blood cell lysis buffer (Solarbio, Beijing, China) for 3 min on ice. After washing twice with cold PBS, the cells were suspended in complete RPMI 1640 medium supplemented with 10% FBS and 1% GlutaMAX and cultured at a concentration of 2 × 10^6^ cells/ml per well in 24-well plates or 5 × 10^6^ cells/ml per well in 96-well plates at 37°C and 5% CO_2_. To induce lymphocyte proliferation, cells were stimulated with ConA (5 *μ*g/ml) or LPS (1 *μ*g/ml). The study was approved by the Animal Ethics Committee of the Third Affiliated Hospital of Sun Yat-sen University ([2019]02-342-01).

### 2.4. Cell Viability and Proliferation Assays

Splenocytes were cultured in 96-well plates at a density of 5 × 10^6^ cells/ml per well and incubated with the indicated concentrations of CAPE for 2 h prior to mitogen stimulation. After 48 h of stimulation by ConA or LPS, 10 *μ*l of CCK-8 solution was added to the medium and incubated for 4 h at 37°C and 5% CO_2_. The absorbance at 450 nm was assessed with a microplate reader (Biotek).

### 2.5. T Cell Culture and Differentiation

For T cell purification, prewarmed (37°C) PBS and RPMI 1640 medium (containing 5% FBS) were used to wash a nylon wool syringe (Polysciences, US) 4-5 times. The purity of T cells was ~85%. Then, 2.0 ml of warm complete RPMI 1640 medium containing 1 − 2 × 10^8^ lymphocytes in suspension was added to the column, and the medium was allowed to drain. An additional 1.0 ml of warm complete RPMI 1640 medium was added to cover the top of the wool and incubated for 1 h at 37°C. Finally, the medium was allowed to drain, and the cells were slowly eluted with warm complete RPMI 1640 medium. Purified T cells (1 × 10^5^ cells/ml) were stimulated with plate-coated anti-CD3 (2 *μ*g/ml) and soluble anti-CD28 (1 *μ*g/ml) monoclonal antibodies (mAbs) for 72 h in the presence or absence of CAPE in 96-well round-bottom plates in a total volume of 200 *μ*l of complete RPMI 1640 medium at 37°C and 5% CO_2_. rIL-12 (10 ng/ml) and an anti-IL-4 mAb (5 *μ*g/ml) were added along with the anti-CD28 mAb to polarize T cells into Th1 cells.

### 2.6. EAE Induction, CAPE Treatment, and Demyelination Scores

Female C57BL/6 mice (6-8 weeks old) were purchased from Vital River Laboratory Animal Technology (Beijing, China) and maintained in the Shaanxi Normal University animal facility under pathogen-free conditions (12-h/12-h light/dark cycle, food and water provided *ad libitum*). Mice were immunized subcutaneously (s.c.) with 100 *μ*g of myelin oligodendrocyte glycoprotein peptide 35-55 (MOG_35-55_) emulsified in complete Freund's adjuvant containing 2 mg/ml *M. tuberculosis* H37 RA on day 0, and 100 ng of pertussis toxin (PT) was administered intraperitoneally (i.p.) on day 0 and day 2. Clinical scores and body weights were recorded daily starting at the beginning of immunization. Mice were housed in accordance with the guidelines of the Shaanxi Normal University Animal Care and Use Committee and were allowed to acclimate for at least 7 days before use.

According to Park et al., significant changes in lymphocyte activities were observed in mice treated with 20 mg/kg CAPE for 14 days [7]. Therefore, in our study, CAPE was given at doses of 20 mg/kg or 40 mg/kg. Mice treated with daily intraperitoneal injections of CAPE were further divided into two groups: the prophylactic group, whose treatment started on the first day of EAE induction, and the therapeutic group, whose treatment started on the first day of symptom onset. The vehicle group received daily intraperitoneal injections of PBS starting on the first day of EAE induction. Each group contained 10 mice. At the end of the study, mononuclear cells were collected from spleens and the CNS after either red blood cell lysis or Percoll separation for flow cytometry analysis.

Clinical scores ranged from 0 to 5 as follows: 0, no signs; 0.5, stiff tail; 1, limp tail; 2, limp tail with gait incoordination; 2.5, paralysis of one hind limb; 3, paralysis of both hind limbs; 3.5, hind limb paralysis and weakness of one forelimb; 4, moribund; and 5, death. Three areas of each lumbar enlargement section (three sections/animal) were graded as follows: score 0, no demyelination; score 1, mild demyelination; score 2, severe demyelination; and score 3, massive demyelination.

### 2.7. Flow Cytometry

Cells were harvested and stained with an anti-CD4 antibody for 30 min at room temperature. After washing with PBS/1% BSA, the cells were incubated with fixation buffer at room temperature in the dark for 20 min and washed with permeabilization (perm) buffer. After removing the supernatant, the cells were resuspended in the perm buffer and incubated at room temperature in the dark for 15 min. Then, the cells were centrifuged, and the pellet was resuspended in 100 *μ*l of perm buffer. The cells were incubated with intracellular antibodies at room temperature in the dark for 30 min. Finally, the cells were washed twice with PBS/1% BSA, resuspended, run on an FACS Canto II flow cytometer (BD Biosciences, San Jose, CA), and analysed by FlowJo (TreeStar, Ashland, OR).

### 2.8. Histopathology and Immunofluorescence

Spinal lumbar enlargements were fixed in 4% paraformaldehyde, embedded in paraffin, and sectioned (4 *μ*m). Some lumbar enlargement sections (3-4 mice per group; 3 sections per animal) were stained with HE to evaluate the degree of inflammatory cell infiltration. Other sections underwent immunofluorescence staining as follows: sections were paraffinized, rehydrated, and blocked with 10% goat serum. Then, the sections were incubated overnight at 4°C with primary antibodies against Iba-1 (1 : 100) and MBP (1 : 200). After washing, the sections were incubated with Alexa Fluor-conjugated secondary antibodies (1 : 300) for 1 h at room temperature, followed by DAPI counterstaining. Images were obtained under a Leica DM 4000 B microscope and analysed through Image J (National Institutes of Health, USA).

### 2.9. Statistical Analysis

Student's *t*-test or the Mann–Whitney *U* test was used to compare data between two groups (*a* = 0.05). Independent nonparametric data with multiple groups, such as clinical scores, were assessed by non-parametric one-way ANOVA (the Kruskal-Wallis test). *P* < 0.05 was considered significant. All statistical analyses were performed using the GraphPad Prism 8.0 software.

## 3. Results

### 3.1. Th1 Polarization Was Prominent in Patients with MS

**Figure 1:**
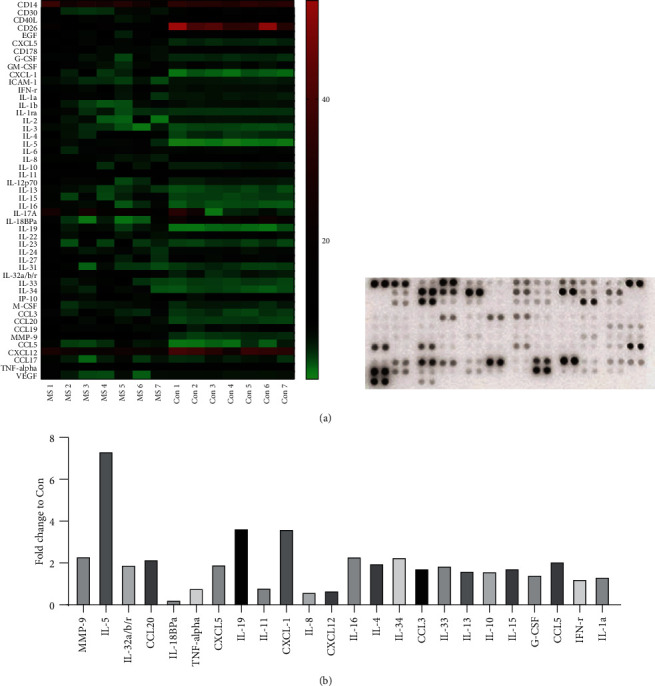
The levels of CSF cytokines and chemokines in patients with relapsing MS (*n* = 7) and patients with noninflammatory disorders (*n* = 7) (a). The fold changes of significantly altered cytokines/chemokines compared to those in the control group (b).

To evaluate the T cell profile in patients with MS, we measured the levels of several cytokines and chemokines in CSF, which can better reflect the inflammatory condition of CNS. The clinical characteristics of the recruited patients are summarized in Table 1. No differences in the CSF protein level or white cell count were found between the MS and control groups.  Figure 1 shows the levels of CSF cytokines/chemokines. The levels of proinflammatory cytokines/chemokines, including MMP-9, IL-32a/b/c, CCL-20, CXCL-5, IL-19, CXCL-1, IL-16, IL-34, CCL-3, IL-15, G-CSF, CCL-5, IFN-*γ*, and IL-1a, as well as the level of one anti-inflammatory cytokine, IL-10, were significantly elevated in the MS patients compared with the controls, and most of these cytokines are associated with Th1 differentiation (e.g., IFN-*γ*, IL-15, and IL-16). Unexpectedly, we observed that the levels of some Th2-type cytokines (IL-4, IL-5, and IL-13) were also higher in the MS group than in the control group, which is in accordance with limited studies [8]. No correlation was observed between significantly altered Th1 and Th2 cytokines.

### 3.2. CAPE Inhibited ConA-Induced T Cell Proliferation and Activation

**Figure 2:**
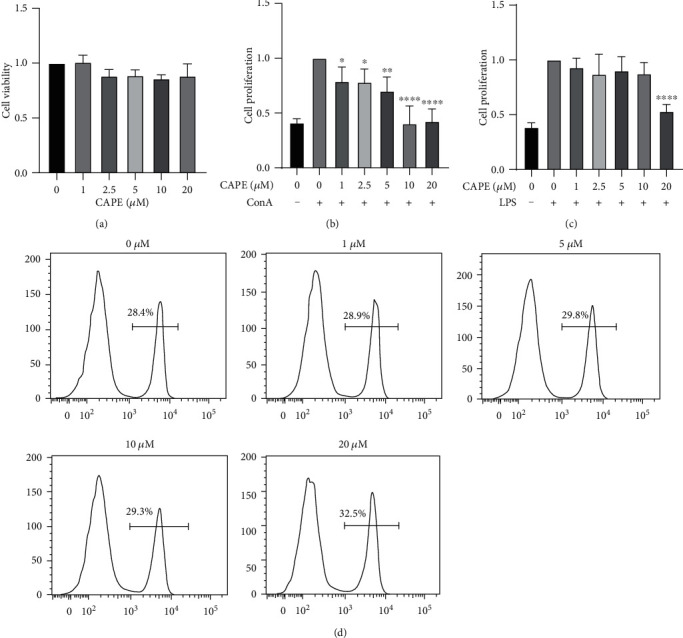
Effects of CAPE on lymphocyte proliferation and CD4^+^ T cell expression. Cells were cultured at a concentration of 5 × 10^6^ cells/ml per well in 96-well plates. To induce lymphocyte proliferation, cells were stimulated with ConA (5 *μ*g/ml) or LPS (1 *μ*g/ml) for 48 h. Cell viability among the groups treated with various concentrations of CAPE (0-20 *μ*M) (a). Cell proliferation of ConA- (b) and LPS-stimulated (c) lymphocytes. The percentage of CD4^+^ T cells in ConA-stimulated splenocytes (d). The data (mean ± SEM) represent three independent experiments. ^∗^*P* ≤ 0.05, ^∗∗^*P* ≤ 0.01, ^∗∗∗∗^*P* ≤ 0.0001 versus the vehicle group.

**Figure 3:**
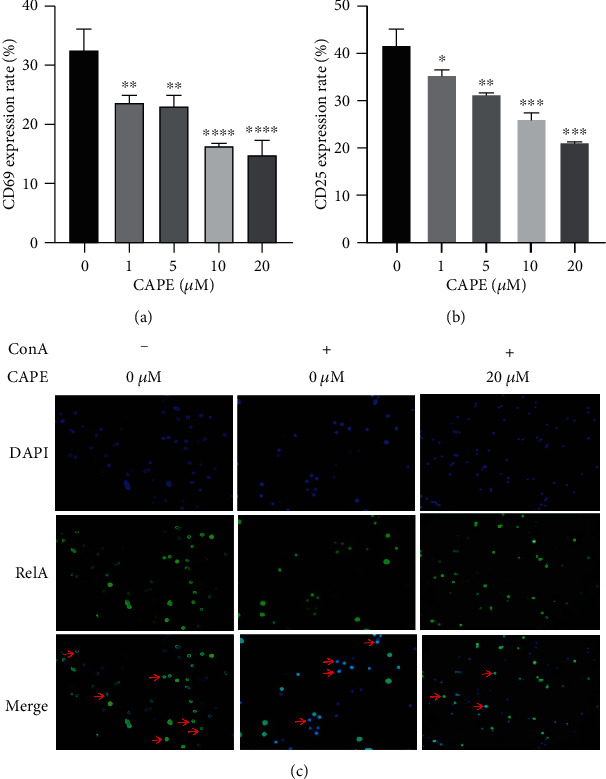
Effects of CAPE on T cell activation and NF-*κ*B p65 nuclear translocation. Lymphocytes were cultured at a concentration of 5 × 10^6^ cells/ml per well in 96-well plates or 2 × 10^6^ cells/ml per well in 24-well plates and stimulated with ConA (5 *μ*g/ml). The percentages of CD69 and CD25 in CD4^+^ T cells after ConA stimulation for 8 h or 24 h (a, b). NF-*κ*B p65 nuclear translocation after stimulating splenocytes with ConA for 2 h (c). The data (mean ± SEM) represent three independent experiments. ^∗^*P* ≤ 0.05, ^∗∗^*P* ≤ 0.01, ^∗∗∗^*P* ≤ 0.001, ^∗∗∗∗^*P* ≤ 0.0001 versus the vehicle group.

As shown in Figure 2(a), cell viability was similar among the groups treated with various concentrations of CAPE for 48 h, indicating that CAPE had no toxicity to splenocytes. To test the effect of CAPE on mitogen-induced cell proliferation, cells were incubated with ConA or LPS, which mainly stimulates T cells or B cells, respectively. CAPE significantly inhibited ConA-stimulated cell proliferation in a dose-dependent manner (Figure 2(b)). In contrast, LPS-induced proliferation was not affected by treatment with 0-10 *μ*M CAPE. A significant change was observed at a concentration of 20 *μ*M CAPE compared to the control group (Figure 2(c)). Since CD4^+^ T cells play a pivotal role in MS, we investigated the frequency of CD4^+^ T cells among ConA-stimulated splenocytes. No differences were detected among cells treated with the indicated doses of CAPE (Figure 2(d)). Upon activation, the expression of several molecules on T cells, such as CD69 (an early activation marker) and CD25 (a middle activation marker), was increased under the regulation of transcription factors, such as NF-*κ*B [9]. Splenocytes were stimulated with ConA for 8 h (for CD69) or 24 h (for CD25). Figures 3(a) and 3(b) indicates that 10 *μ*M CAPE pretreatment significantly suppressed not only the percentage of cells expressing CD69 and CD25 but also the percentage of CD4^+^CD69^+^ and CD4^+^CD25^+^ cells. The transcriptional activity of these activation molecule genes has been reported to be highly dependent on NF-*κ*B [9]. Consistent with this report, nuclear translocation of NF-*κ*B p65 was inhibited by 20 *μ*M CAPE treatment after stimulation with ConA for 2 h.

### 3.3. CAPE Suppressed IFN-*γ* Expression and Improved Foxp3 Expression in Naive CD4^+^ T Cells

**Figure 4:**
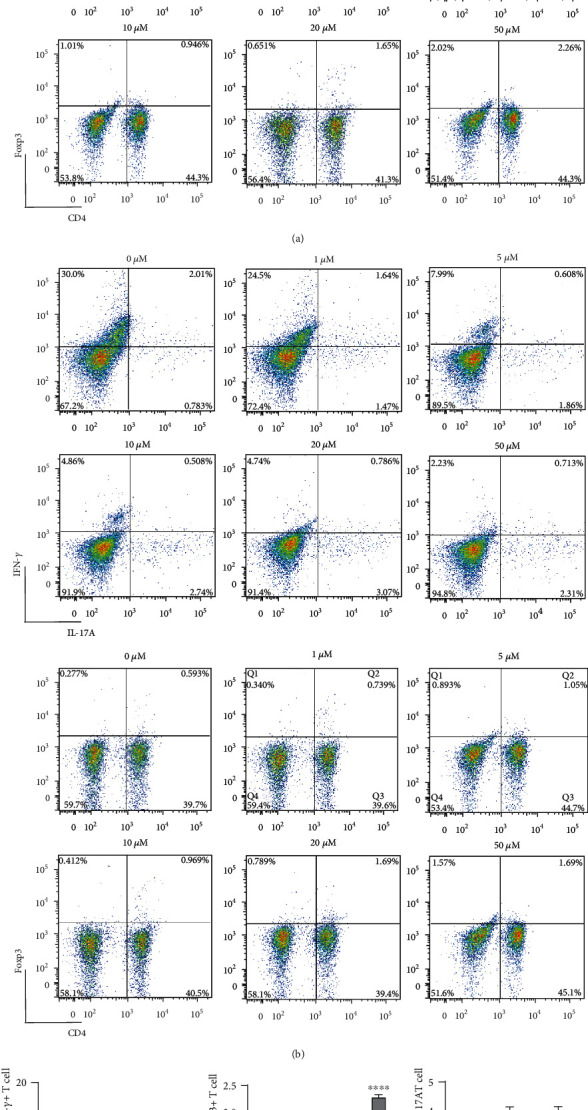
Effects of CAPE on T cell differentiation in Th0- (a, c) and Th1-polarizing (b, d) conditions. Purified T cells (1 × 10^5^ cells/ml) were stimulated with plate-coated anti-CD3 (2 *μ*g/ml) and soluble anti-CD28 (1 *μ*g/ml) monoclonal antibodies for 72 h in the presence or absence of CAPE in 96-well plates. rIL-12 (10 ng/ml) and an anti-IL-4 mAb (5 *μ*g/ml) were added along with the anti-CD28 mAb to polarize T cells into Th1 cells. The data (mean ± SEM) represent three independent experiments. ^∗^*P* ≤ 0.05, ^∗∗^*P* ≤ 0.01, ^∗∗∗^*P* ≤ 0.001, ^∗∗∗∗^*P* ≤ 0.0001 versus the vehicle group.

To evaluate the effects of CAPE on CD4^+^ T cells, we detected the levels of IFN-*γ*, IL-17A, and Foxp3 by flow cytometry. As shown in Figures 4(a) and 4(c), CAPE substantially inhibited the level of IFN-*γ* induced after T cell stimulation with anti-CD3 and anti-CD28 antibodies for 72 h. In addition, CAPE treatment increased Foxp3 expression in cells at a concentration of 20 *μ*M. No difference was observed in the percentage of CD4^+^ T cells or CD4^+^IL-17A^+^ T cells between CAPE- and vehicle-treated cells. Since Th1 cells were significantly increased in the MS patients compared to the controls, we established Th1-polarizing conditions with rIL-12 and an anti-IL-4 mAb. Figures 4(b) and 4(d) show that CAPE significantly inhibited IFN-*γ* (*P* < 0.0001) but increased Foxp3 (*P* < 0.0001) levels in CD4^+^ T cells. CD4^+^ T cells and CD4^+^IL-17A^+^ T cell numbers were similar between CAPE- and vehicle-treated T cells. These results strongly indicated that the anti-inflammatory functions of CAPE were mediated at least partly through a direct inhibitory effect on the differentiation of the Th1 and Treg subsets, which may alleviate EAE *in vivo*.

### 3.4. Prophylactic CAPE Treatment Reduced EAE Severity

**Figure 5:**
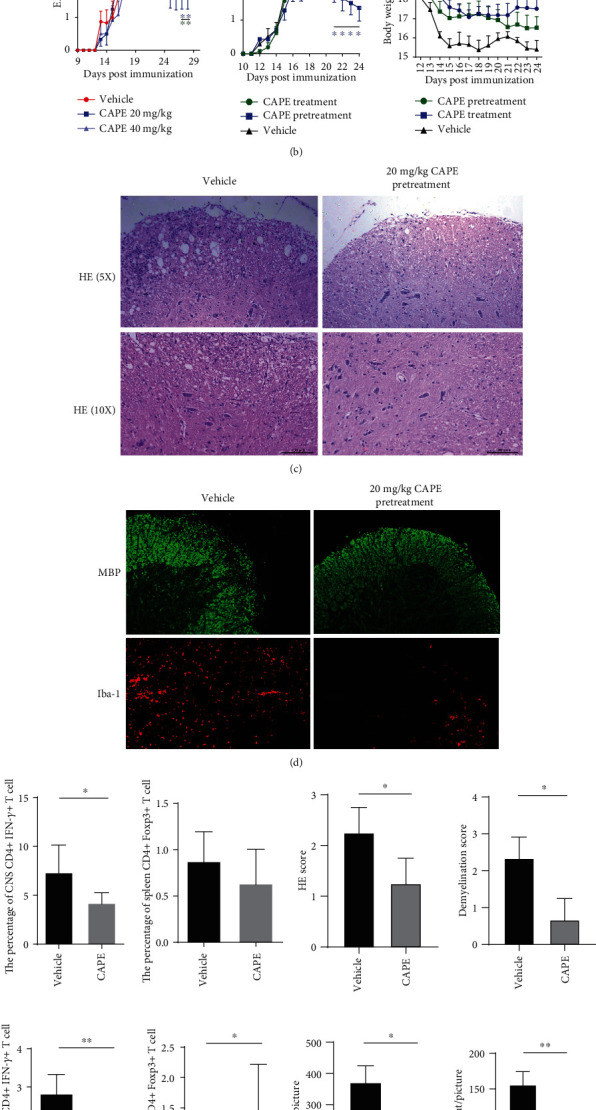
Therapeutic efficacy of CAPE in MOG_35-55_-induced EAE mice. Mice were immunized with MOG_35-55_ peptide emulsified in complete Freund's adjuvant. Daily CAPE administration was initiated on the day of immunization (pretreatment) or on the day of symptom onset (treatment). Body weights and EAE scores were evaluated starting on the day of immunization. Different regimens of CAPE administration in EAE mice (a). The impacts of different regimens and doses on the course of EAE (b). Inflammatory infiltration assessed by HE staining (c), microglia/microphage activation assessed by Iba-1, and demyelination demonstrated by MBP (d) in EAE mice. HE scores, demyelination scores, cell infiltration, and the proportion of T cells in EAE mice (e). Each group contained 10 mice. Data are presented as the mean ± SEM. ^∗^*P* ≤ 0.05, ^∗∗^*P* ≤ 0.01, ^∗∗∗^*P* ≤ 0.001, ^∗∗∗∗^*P* ≤ 0.0001 versus the vehicle group.

To investigate the effect of CAPE on EAE, we administered CAPE in a prophylactic or therapeutic treatment regimen and monitored disease severity (Figure 5(a)). Prophylactic CAPE groups were initially treated with 20 or 40 mg/kg. We observed reductions in EAE scores at both concentrations, with no further improvement at 40 mg/kg (Figure 5(b)). CAPE at 20 mg/kg was therefore used in the subsequent experiments. Administration of CAPE starting on day 0 showed profound effects in terms of a reduced disease incidence and significantly reduced clinical scores from day 21 onward. More relevant to human MS management, therapeutic administration of CAPE subsequent to the appearance of clinical symptoms produced no benefits in EAE. Body weights were much lower in the vehicle group than in the CAPE pretreatment group and were slightly higher in the CAPE treatment group (Table 2; Figure 5(b)).

At the end of the experiment, mononuclear cells were collected from spleens and the CNS from mice treated with CAPE and vehicle under a prophylactic regimen, and the percentages of different T cell lineages were determined. Consistent with the *in vitro* findings, CAPE treatment significantly reduced the level of Th1 cells in both the spleen and the CNS, and increased Treg numbers were found in the CNS (Figure 5(e)). Compared to the vehicle group, the CAPE treatment group exhibited milder inflammatory cell and microglia/macrophage infiltration around the spinal cord (Figures 5(c) and 5(d)). In addition, demyelination and HE scores were significantly lower in the CAPE treatment group than in the vehicle group (Figure 5(e)).

## 4. Discussion

Our present study showed the therapeutic potential of CAPE in alleviating disease onset and severity in an EAE model through Th1 infiltration inhibition, macrophage/microglia activation, and leukocyte recruitment in the CNS. Consistently, a previous study by Ilhan et al. demonstrated that CAPE can protect against EAE and decrease oxidative tissue damage by blocking reactive oxygen species (ROS) production and NF-*κ*B activation [10]. Activated macrophages and microglia are the main sources of ROS in the CNS, thereby playing a crucial role in aggravating the immune response and tissue injury. In our study, CAPE treatment efficiently inhibited the activation of these cells, which helps explain the therapeutic value reported by Ilhan et al. In addition, CAPE has been found to downregulate the secretion of inducible nitric oxide synthase (iNOS), cyclooxygenase-2, and nitric oxide in LPS-stimulated microglia *in vitro* [11], suggesting that CAPE may be a potent antioxidant.

Comparison of CSF cytokine/chemokine levels between patients with relapsing MS and other noninflammatory diseases revealed that the level of IFN-*γ*, a typical Th1 cytokine, was significantly increased in the MS group. However, the levels of Th17 cytokines, such as IL-17, IL-21, and IL-22, were not increased, which is consistent with previous findings [12–14]. In a recent report, IFN-*γ* was found to be increased in CSF from patients with MS, whereas increased IL-17 was detected in serum [14], suggesting that the Th1 response plays a more critical role in orchestrating the MS immunopathological cascade. Since CD4^+^ T cells can differentiate into various subsets with distinct immune capacities, downregulating the frequencies of pathogenic Th1/Th17 populations while maximally reversing anti-inflammatory subsets is a reasonable strategy when treating MS [15]. Our *in vitro* and *in vivo* findings indicated that CAPE was able to decrease the percentage of Th1 cells without altering the percentages of CD4^+^ T cells and Th17 cells. Consistently, Choi et al. observed that CAPE significantly reduced the level of IFN-*γ*, whereas Th17 and Th2 cytokines were not affected in an experimental autoimmune uveoretinitis model [8]. By contrast, the study by Wang et al. demonstrated that CAPE inhibited not only IFN-*γ* production in CD4^+^ T cells stimulated by CD3 and CD28 antibodies but also IL-5 in both healthy subjects and asthma patients [16]. In our *in vitro* findings, under both Th0- and Th1-polarizing conditions, CAPE administration markedly improved the proportion of CD4^+^Foxp3^+^ T cells *in vitro*. In the EAE model, the frequency of this T cell population, which was referred to as Tregs, was also upregulated in the CNS. Our results highlight the importance of CAPE in modulating T cell activation and differentiation, which may help in the management of MS patients. Further efforts are required to investigate the exact mechanism by which CAPE modulates these T cell subsets.

Consistent with earlier findings [9], CAPE effectively inhibited ConA-stimulated T cell proliferation *in vitro*. Interestingly, one study reported that T cell proliferation induced by ConA was significantly increased in mice treated with 20 mg/kg CAPE for two weeks [11]. Furthermore, an increase in the ratio of CD4^+^/CD8^+^ T cells was noted in the CAPE-treated group compared to the vehicle group [9], which conflicts with our findings and those of other groups [7]. We assume that the discrepancy may be attributable to the basic state of the studied mice. Evaluating the impact of CAPE on immune cells in healthy animals would be interesting. Additionally, activation of T cells induced by TCR and costimulatory molecules immediately promotes the activity of many transcription factors, including the NF-*κ*B family [17]. Increasing evidence has demonstrated that inhibiting NF-*κ*B might improve EAE by altering peripheral and CNS T cell infiltration, impairing T cell proliferation, and skewing T cells towards a non-Th1/Th17 phenotype [18–20]. In addition, many natural compounds have been identified to at least partly target the NF-*κ*B pathway and have exhibited promising therapeutic potential in treating EAE [21, 22]. Our data indicated that CAPE administration reduced the nuclear translocation of NF-*κ*B p65. In addition to NF-*κ*B, the nuclear factor of activated T cells (NFAT), which is involved in regulating gene expression, including IFN-*γ*, was recently found to be a target of CAPE by Marquez et al. [9].

Our study has several limitations. First, suppressing T cell activity in healthy organisms may lead to negative effects such as persistent infection, cancer, and autoimmunity. Therefore, whether the normal immune responses of other organs are impaired during CAPE treatment should be considered at the same time. Second, although the immunofluorescence data indicated that CAPE likely inhibits the nuclear translocation of NF-*κ*B p65, Western blot will be a better method to confirm this conclusion. Third, the effects of CAPE on unstimulated lymphocytes/T cells require further investigation.

In conclusion, our results indicate that CAPE exhibits strong anti-inflammatory and immunomodulatory effects by suppressing NF-*κ*B activation and T cell activity in EAE, implying the possibility of using CAPE as an immunomodulatory agent for MS treatment.

## Figures and Tables

**Table 1:** Clinical characteristics of the patients with relapsing MS and noninflammatory disorders.

	MS (*n* = 7)	Control (*n* = 7)
Gender ratio (*F*/*M*)	5/2	4/3
Age at onset (*y*)	24 (15-32)	33 (20-47)
Disease course (*m*)	27.5 (7-72)	—
EDSS at sampling	3.25 (2.5-5)	—
EDSS at the last visit	1 (1-2.5)	—
CSF WBC (/*μ*l)	2 (0-20)	1 (0-4)
CSF protein (mg/L)	0.19 (0.12-0.30)	0.14 (0.07-0.34)
Abnormal spinal MRI, *n* (%)	4/7 (57)	0/7 (0)
Treatment, *n* (%)		—
Azathioprine	3/7 (43)	
Interferon-*β*	1/7 (14)	
Terflutamide	1/7 (14)	
Oral methylprednisolone	2/7 (29)	

**(a):** 

	Vehicle	CAPE 20 mg/kg	CAPE 40 mg/kg
Disease incidence	100%	60%	60%
Death	0%	0%	0%
Disease onset (*d*)	15.0 ± 2.0	14.5 ± 1.2	14.5 ± 1.5
EAE score at the peak time	3.1 ± 0.7	2.8 ± 0.7	2.7 ± 0.5
EAE score at sampling	2.8 ± 0.7	1.58 ± 0.8^∗^	1.58 ± 0.9^∗^

**(b):** 

	Vehicle	CAPE pretreatment	CAPE treatment
Disease incidence	89%	54%^∗^	88%
Death	0%	0%	0%
Disease onset (*d*)	14.1 ± 2.5	13.3 ± 1.3	14.4 ± 1.6
EAE score at the peak time	2.9 ± 1.4	2.4 ± 1.0	2.8 ± 0.8
EAE score at sampling	2.8 ± 0.8	1.4 ± 1.0^∗^	2.3 ± 1.0

## Data Availability

The research data used to support the findings of this study are included within the article (tables, figures).

## Supplementary Materials

Supplementary Materials The chemical structure of CAPE.
