# Supplementary figures and images for: Caffeic Acid Phenethyl Ester Protects against Experimental Autoimmune Encephalomyelitis by Regulating T Cell Activities
Source: Oxid Med Cell Longev. 2020 Oct 9;2020:7274342. doi: 10.1155/2020/7274342 (PMC7568814; doi:10.1155/2020/7274342)

1. The chemical structure of CAPE

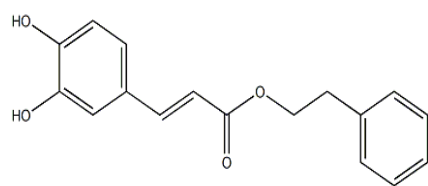

Supplement: Supplementary Materials — The chemical structure of CAPE. [file 7274342.f1.pdf]
